# A novel *HMBS* gene mutation in acute intermittent porphyria: a case report of abdominal pain, seizures, and reversible neuroimaging findings

**DOI:** 10.3389/fgene.2025.1551832

**Published:** 2025-03-05

**Authors:** Wentao Dong, Bingliang Zeng, Xiaolian Wang, Rui Zhang, Pei Huang, Bing Fan, Min Yuan, Zicong Li

**Affiliations:** ^1^ Department of Radiology, Jiangxi Provincial People’s Hospital, The First Affiliated Hospital of Nanchang Medical College, Nanchang, China; ^2^ Medical College of Nanchang University, Nanchang University, Nanchang, China; ^3^ Department of Neurology, Jiangxi Provincial People’s Hospital, The First Affiliated Hospital of Nanchang Medical College, Nanchang, China

**Keywords:** HMBS gene mutation, abdominal pain, seizures, neuroimaging, acute intermittent porphyria

## Abstract

**Background:**

Acute intermittent porphyria (AIP) is a rare metabolic disorder resulting from defects in the heme biosynthesis pathway, often presenting with non-specific symptoms such as abdominal pain, seizures, and neuropsychiatric disturbances. Diagnosis is challenging due to the overlap of symptoms with other conditions, and early recognition is critical for effective treatment.

**Case Presentation:**

A 24-year-old female presented with a 6-day history of persistent lower abdominal pain and generalized tonic-clonic seizures, following the consumption of seafood. Neuroimaging revealed white matter hyperintensities, and urine analysis showed dark red discoloration, suggestive of porphyria. Genetic testing confirmed a novel c.499-1_514del mutation in the *HMBS* gene, diagnosing AIP. The patient was treated with intravenous glucose, heme arginate, and anticonvulsants. Symptom resolution was noted within days, and follow-up MRI showed significant improvement.

**Conclusion:**

This case underscores the importance of early diagnosis and management in AIP. Genetic testing plays a crucial role in confirming the diagnosis, especially in atypical cases. Timely intervention with glucose and heme arginate, combined with supportive care, led to rapid symptom resolution, reinforcing the reversibility of AIP-associated neuroimaging changes. Clinicians should maintain a high index of suspicion for AIP in patients with unexplained abdominal and neurological symptoms to prevent long-term complications.

## Introduction

Acute intermittent porphyria (AIP) is the most common of the acute porphyrias and part of a group of metabolic disorders caused by defects in the heme biosynthesis pathway. AIP is specifically associated with mutations in the *HMBS* gene (hydroxymethylbilane synthase), leading to reduced enzyme activity and the accumulation of toxic precursors, primarily delta-aminolevulinic acid (ALA) and porphobilinogen (PBG) (Souza et al., 2021). The incidence of AIP varies globally, estimated at 1 in 20,000 in certain populations but remains underdiagnosed due to its non-specific presentation (Lefever et al., 2022; Muschalek et al., 2022). The pathophysiology of AIP involves a cascade of biochemical disturbances triggered by various factors, including medications, hormonal changes, dietary triggers, and stress (Phillips, 2019; Spiritos et al., 2019).

Clinical manifestations of AIP are diverse and can include severe abdominal pain, often misattributed to gastrointestinal or gynecological causes, and neuropsychiatric symptoms ranging from seizures and peripheral neuropathy to altered mental status (Kizilaslan et al., 2023). Neurological complications can be severe, resulting in long-term deficits if not promptly recognized and treated (Ramanujam and Anderson, 2015; Balwani et al., 2024). Neuroimaging findings in AIP may show changes consistent with metabolic encephalopathy, such as white matter hyperintensities on T2-weighted and FLAIR MRI sequences. The condition is also associated with posterior reversible encephalopathy syndrome (PRES), which reflects the reversible nature of the underlying pathophysiological changes when managed effectively (Yang et al., 2020; Jaramillo-Calle et al., 2019).

This case report explores a young female patient presenting with characteristic AIP symptoms and demonstrates how timely diagnosis, supported by genetic and neuroimaging studies, can lead to successful management and outcome.

## Case Presentation

A 24-year-old female presented with a 6-day history of persistent lower abdominal pain, which began following the consumption of a seafood buffet. Two days after the onset of abdominal pain, she developed recurrent limb convulsions, followed by generalized tonic-clonic seizures. Seizures were characterized by generalized tonic-clonic movements, including limb convulsions, eye deviation, frothing at the mouth, and postictal confusion with transient memory loss.

The patient had no prior medical history of seizures, neurological disorders, or metabolic conditions. There was no known family history of porphyria. Possible triggering factors included dietary intake, stress, and hormonal changes. She denied any recent medication use, alcohol consumption, or toxin exposure.

Upon admission, a thorough physical and neurological examination was performed. Mental status assessment revealed confusion and disorientation, particularly postictally, with mild amnesia. The patient was alert but had difficulty recalling recent events. Motor function showed no signs of hemiparesis or ataxia, but mild weakness in both upper limbs was noted after the seizures. Reflexes were normal, with no hyperreflexia or signs of pathological reflexes. Cranial nerve assessment was unremarkable, with no facial asymmetry or abnormalities in visual fields, pupillary reflexes, or extraocular movements.

**FIGURE 1:**
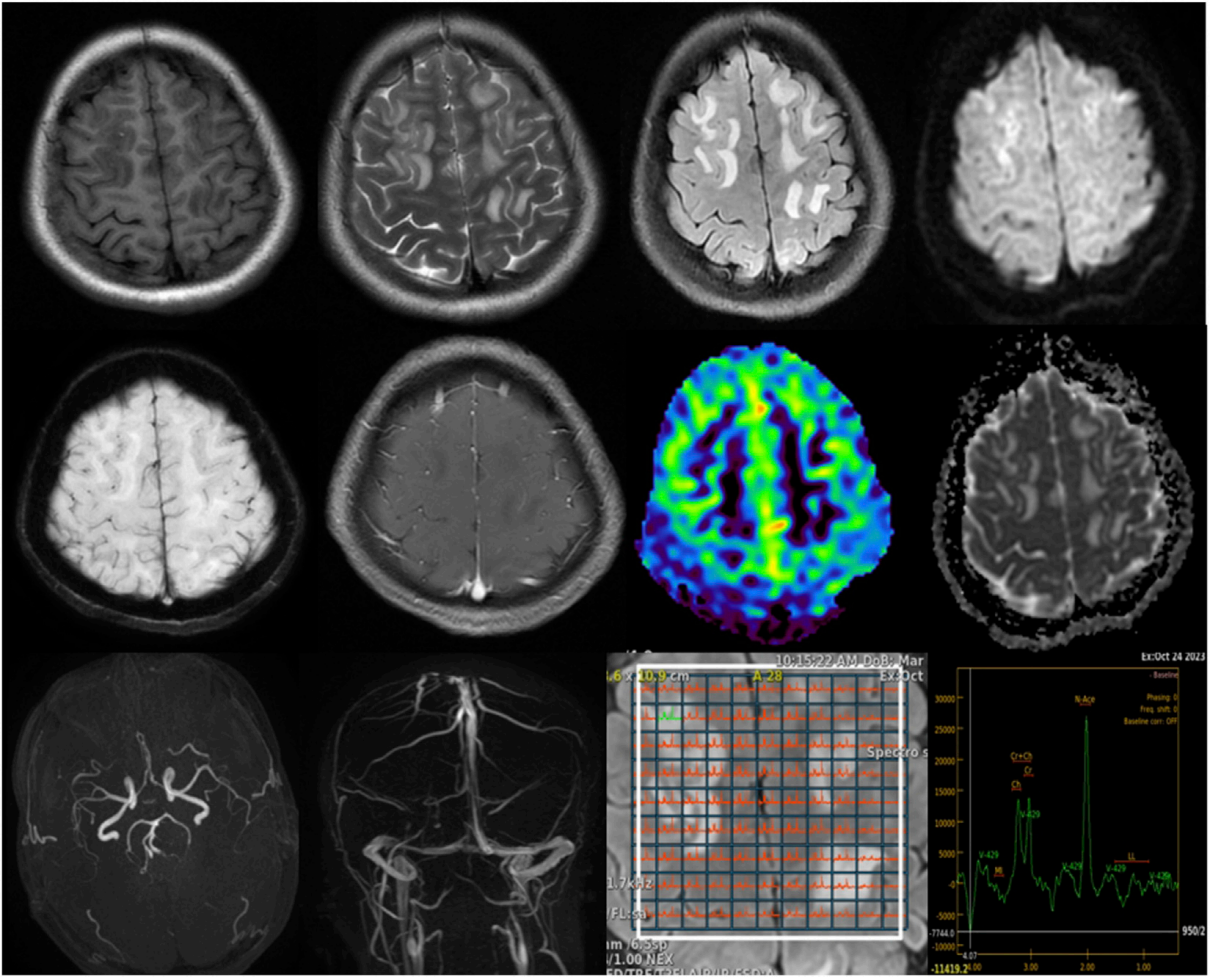
Brain MRI findings: T1-weighted images show slightly hypointense to isointense lesions, while T2-weighted and FLAIR sequences reveal hyperintense lesions predominantly involving the bilateral cerebral white matter with asymmetric distribution. DWI demonstrates high signal intensity, with corresponding high values on the apparent diffusion coefficient ADC map, indicating vasogenic edema. SWI shows no significant susceptibility artifacts. Post-contrast images reveal mild to moderate enhancement. MRS, MRA, and MRV findings are unremarkable, as shown.

Initial abdominal ultrasound and contrast-enhanced CT scans were inconclusive, revealing only a suspected right adnexal corpus luteum cyst. Given the persistence of neurological symptoms, a brain MRI was subsequently performed for further evaluation. Imaging revealed slightly prolonged T1 and T2 signals with high signals on FLAIR sequences. The lesions were predominantly located in the bilateral cerebral hemispheres and the right cerebellar hemisphere, displaying an asymmetric distribution and primarily affecting the white matter. Diffusion-weighted imaging (DWI) showed high signal intensity, while the apparent diffusion coefficient (ADC) map indicated high values. No significant low-signal areas were noted on susceptibility-weighted imaging (SWI). Post-contrast images displayed mild to moderate enhancement, while magnetic resonance spectroscopy (MRS), arterial spin labeling (ASL), magnetic resonance angiography (MRA), and magnetic resonance venography (MRV) all showed normal findings. Based on these imaging characteristics (Figure 1) and neurological symptoms, initial considerations included encephalitis, demyelinating processes, or metabolic/toxic encephalopathy.

Laboratory investigations revealed elevated creatine kinase (CK) levels at 14,538 U/L (normal range: 24–195 U/L), gamma-glutamyl transferase (GGT) at 370 U/L (normal range: 9–48 U/L), suggesting significant muscle injury and potential liver or biliary stress, respectively. Sodium was low at 130 mmol/L (normal range: 135–145 mmol/L), while potassium, calcium, magnesium, and phosphorus were within normal ranges, supporting the overall metabolic stability aside from mild hyponatremia. Additionally, inflammatory markers such as C-reactive protein (CRP) and erythrocyte sedimentation rate (ESR) were within normal ranges, suggesting the absence of a significant inflammatory response. Renal function was assessed, revealing a serum creatinine level of 71.3 μmol/L (normal range: 50–100 μmol/L) and a blood urea nitrogen level of 5.22 mmol/L (normal range: 2.5–7.1 mmol/L), both of which indicate stable kidney function without signs of impairment. Uric acid was slightly elevated at 419 μmol/L (normal range: 120–400 μmol/L), which may suggest a metabolic component, but does not directly indicate renal impairment. Urine analysis showed notable findings: urobilinogen 2+, while urine occult blood, glucose, ketones, bilirubin, and white blood cells were all negative. The presence of urobilinogen at this level may suggest mild liver involvement or hemolysis. Due to these findings, a multidisciplinary team discussion was held to consider differential diagnoses. Rhabdomyolysis was considered as a potential cause given the elevated CK levels. However, AIP could not be ruled out. Consequently, it was recommended to conduct specialized genetic testing for porphyria, which was sent out to an external laboratory for confirmation.

**FIGURE 2:**
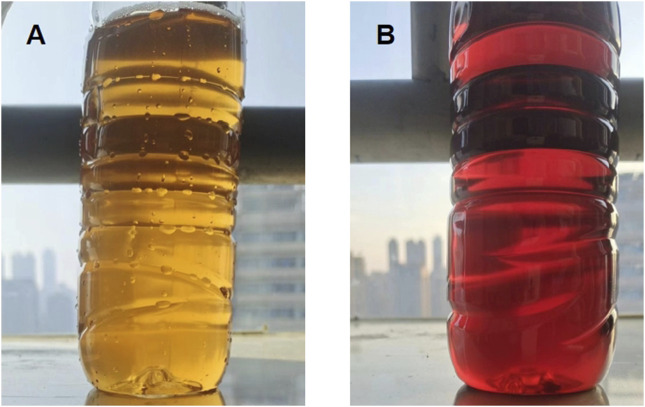
Urine color changes in AIP: **(A)** Freshly collected urine sample, showing a typical yellow color. **(B)** Same urine sample after standing for 6 h, showing a characteristic dark red color due to porphyrin precursor accumulation.

**FIGURE 3:**
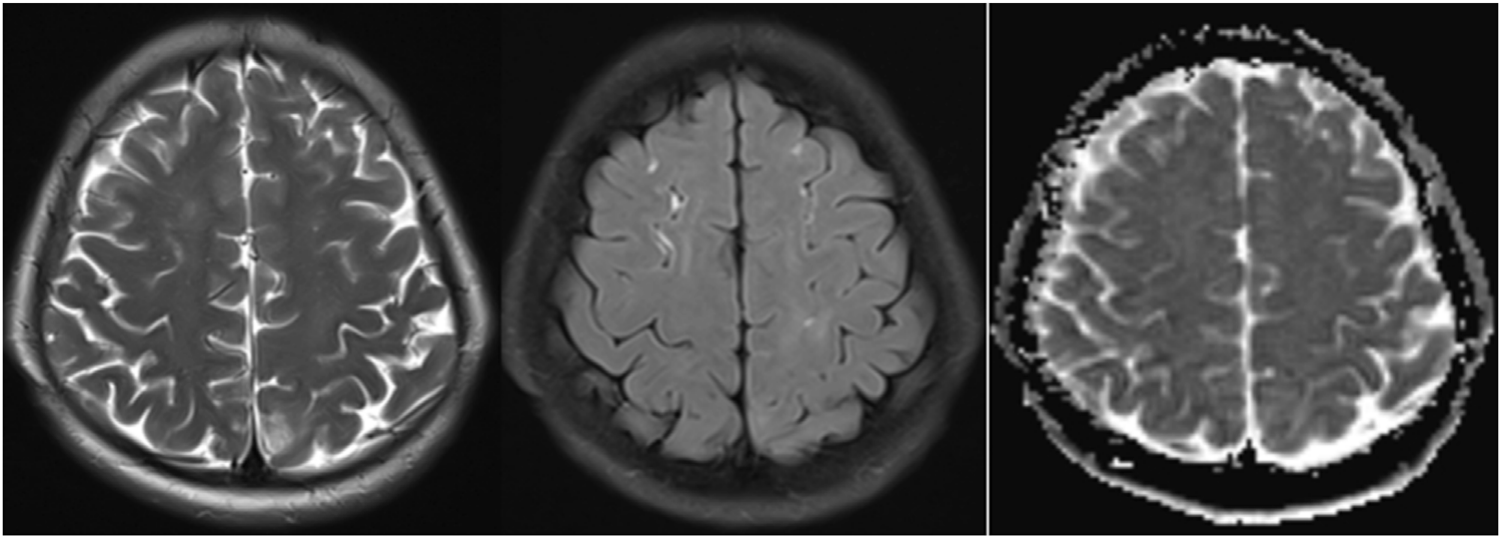
MRI findings after 2 weeks of treatment in AIP: Significant reduction in hyperintense lesions on T2-weighted, FLAIR sequences and ADC map.

To explore the potential for AIP, urine analysis was performed, revealing a notable color change from light yellow to dark red upon standing for six hours—an indication of porphyrin precursor accumulation (Figure 2). However, levels of ALA and PBG in the urine were not measured in this case, as this test was not performed during the patient’s initial workup. Genetic testing was conducted by a certified external laboratory using next-generation sequencing (NGS), which identified the c.499-1_514del mutation in the *HMBS* gene. This finding, documented in the official diagnostic report, confirmed the diagnosis of AIP. Follow-up MRI 2 weeks post-treatment showed significant resolution of these hyperintensities, suggesting the reversible nature of AIP-associated cerebral changes (Figure 3).

## Management and outcome

The patient was treated with intravenous glucose and heme arginate to suppress hepatic ALA synthesis. Intravenous glucose was administered as a 10% dextrose infusion at a rate of 200–500 mL/h, titrated based on blood glucose levels to maintain euglycemia. Heme arginate was administered at a dose of 3 mg/kg body weight IV once daily for 4 consecutive days, diluted in 100 mL of 0.9% normal saline and infused over 30 min. Levetiracetam was administered as an anticonvulsant at a dose of 500 mg to 1,000 mg IV twice daily, adjusted based on the patient’s response and renal function.

In addition to the specific treatments, supportive treatment included correction of hyponatremia with intravenous sodium chloride solution (normal saline), as well as monitoring and adjusting electrolytes (potassium, calcium, and magnesium) to maintain metabolic stability. Fluid management was provided to ensure adequate hydration, and pain management was addressed with analgesics as necessary. The patient’s symptoms gradually improved, with limb seizures resolving within 2 days and abdominal pain significantly decreasing by day five. No further generalized seizures occurred, and the patient was discharged in stable condition. Follow-up MRI confirmed the near-complete resolution of the previously noted abnormalities, reinforcing the effectiveness of early intervention in reversing AIP-associated neuroimaging changes.

## Discussion

AIP is a rare metabolic disorder resulting from defects in the heme biosynthesis pathway, primarily due to mutations in the *HMBS* gene (Li et al., 2023; Bustad et al., 2021). This case report highlights several distinctive features, most notably the novel pathogenic mutation identified in the *HMBS* gene (c.499-1_514del variant). This mutation has not been previously reported in the literature, marking an important contribution to the genetic spectrum of AIP. The c.499-1_514del mutation is likely to lead to the loss of function of hydroxymethylbilane synthase, an enzyme involved in heme synthesis, based on its location in the *HMBS* gene and the known effects of similar mutations. As a result, the accumulation of porphyrin precursors such as ALA and PBG occurs, which is likely responsible for the neurotoxic symptoms seen in this patient. This discovery not only adds a new genetic variation to the AIP gene pool but also highlights the importance of genetic testing in diagnosing this rare condition, especially in atypical cases. The identification of this novel mutation has significant implications for family screening and early diagnosis, which could improve outcomes by facilitating early intervention and management (Wang et al., 2023).

In the differential diagnosis, we systematically ruled out several potential causes for the patient’s symptoms. Initially, encephalitis was suspected given the neurological symptoms; however, the MRI findings, which predominantly involved white matter lesions, did not support this diagnosis, as encephalitis usually affects gray matter regions (Guo et al., 2022; Qiao et al., 2020). Additionally, inflammatory markers were unremarkable, further ruling out this possibility. Demyelinating conditions such as multiple sclerosis were also considered, but the asymmetric distribution of lesions was atypical for such processes (Wattjes et al., 2021; Benedict et al., 2020). Metabolic or toxic encephalopathy was another differential, but the normal metabolic panel and the imaging pattern, which showed vasogenic rather than cytotoxic edema, helped exclude this possibility (de Oliveira et al., 2019). Rhabdomyolysis was initially considered due to elevated CK levels; however, the absence of myoglobinuria, muscle pain, and renal dysfunction argued against this diagnosis (Stahl et al., 2020).

Neuroimaging in AIP typically reveals white matter hyperintensities on T2-weighted and FLAIR MRI sequences (Kumar et al., 2023), as was observed in this case. These findings are often transient and can mimic conditions like PRES. The high signal intensity seen on DWI and high ADC values further supported the diagnosis of vasogenic edema, which is a reversible condition typically associated with endothelial dysfunction and disruption of the blood-brain barrier (Ando et al., 2022; Anderson et al., 2020). The absence of restricted diffusion on DWI and the normalization of ADC values after treatment provided additional evidence of the reversible nature of the cerebral lesions. The significant improvement in the patient’s MRI findings 2 weeks post-treatment with heme arginate and glucose infusion underscores the reversibility of the neuroimaging changes seen in AIP, emphasizing the importance of early diagnosis and treatment to prevent long-term neurological sequelae.

The clinical presentation of AIP can be challenging, as it manifests with a wide range of symptoms that often overlap with other conditions. In this case, the patient presented with severe, recurrent abdominal pain and generalized tonic-clonic seizures, both of which are hallmark symptoms of AIP. These symptoms are typically triggered by the accumulation of toxic porphyrin precursors, such as ALA and PBG, which affect the central nervous system. Seizures in AIP can be precipitated by electrolyte imbalances, particularly hyponatremia, as seen in this patient. The abnormal sodium levels (130 mmol/L) in the patient were likely caused by inappropriate antidiuretic hormone secretion (Yusuf et al., 2023), which is common in AIP and exacerbates the neurological symptoms. In this case, the initial diagnosis of a right adnexal corpus luteum cyst highlighted the diagnostic challenges posed by non-specific presentations.

Laboratory investigations play a crucial role in diagnosing AIP. Elevated CK levels, as observed in this patient, indicate significant muscle involvement, which may be related to the neurological effects of AIP. Hyponatremia is another common feature, often overlooked in AIP, but it can exacerbate the neurological symptoms and contribute to the onset of seizures. Urine analysis revealed a characteristic darkening of the urine upon standing, which is a classic diagnostic feature of AIP due to the oxidation of porphyrin precursors (Storjord et al., 2023). This visual clue is crucial in raising suspicion for AIP and prompting further genetic testing. The diagnosis was ultimately confirmed through genetic analysis, which identified the novel c.499-1_514del mutation in the *HMBS* gene. This mutation leads to a deficiency of hydroxymethylbilane synthase, impairing the heme biosynthesis pathway and contributing to the accumulation of porphyrin precursors.

The integration of clinical, biochemical, genetic, and imaging findings is essential for accurate diagnosis and management. In this case, treatment with intravenous glucose and heme arginate effectively suppressed the production of porphyrin precursors, stabilizing the patient’s condition. The follow-up MRI confirmed the near-complete resolution of the previously noted abnormalities, reinforcing the effectiveness of early intervention in reversing AIP-associated neuroimaging changes. The use of anticonvulsants, such as levetiracetam, helped prevent further seizures, and supportive care addressed electrolyte imbalances, particularly the hyponatremia.

This case underscores the complexity of diagnosing AIP, especially when clinical symptoms overlap with other more common conditions. The discovery of a novel mutation in the *HMBS* gene adds to the growing body of knowledge about the genetic underpinnings of AIP and highlights the importance of genetic testing in cases with atypical presentations. The rapid improvement in neuroimaging findings following treatment underscores the importance of early recognition and intervention in preventing long-term complications. Given the rarity of AIP and the potential for reversible neurological changes, it is crucial for clinicians to consider AIP in the differential diagnosis of young adults presenting with unexplained neurovisceral symptoms.

## Conclusion

AIP remains a challenging diagnosis due to its non-specific clinical manifestations and the overlap of symptoms with other more common conditions. This case highlights the importance of early recognition and appropriate management of AIP, particularly in young adults presenting with unexplained neurovisceral symptoms such as abdominal pain and seizures. The identification of a novel pathogenic mutation in the *HMBS* gene further contributes to the expanding genetic spectrum of AIP and underscores the role of genetic testing in the diagnosis of atypical cases. Timely intervention with intravenous glucose and heme arginate, along with supportive care, resulted in a rapid resolution of both neurological and visceral symptoms, demonstrating the reversible nature of AIP-associated neuroimaging changes. This case reinforces the need for clinicians to maintain a high index of suspicion for AIP in patients with recurrent abdominal pain, neurological symptoms, and unexplained imaging findings, as early treatment can prevent long-term neurological sequelae and improve patient outcomes.

## Data Availability

The original contributions presented in the study are included in the article/supplementary material, further inquiries can be directed to the corresponding authors.

## Abbreviations

AIP, Acute intermittent porphyria; ALA, delta-aminolevulinic acid; PBG, porphobilinogen; PRES, posterior reversible encephalopathy syndrome; DWI, Diffusion-weighted imaging; ADC, apparent diffusion coefficient; SWI, susceptibility-weighted imaging; MRS, magnetic resonance spectroscopy; ASL, arterial spin labeling; MRA, magnetic resonance angiography; MRV, magnetic resonance venography; CK, creatine kinase; GGT, gamma-glutamyl transferase; CRP, C-reactive protein; ESR, erythrocyte sedimentation rate; *HMBS*, hydroxymethylbilane synthase.
